# Influence of deep learning image reconstruction algorithm for reducing radiation dose and image noise compared to iterative reconstruction and filtered back projection for head and chest computed tomography examinations: a systematic review

**DOI:** 10.12688/f1000research.147345.1

**Published:** 2024-04-15

**Authors:** Obhuli Chandran M, Saikiran Pendem, Priya P S, Cijo Chacko, Priyanka -, Rajagopal Kadavigere

**Affiliations:** 1 Department of Medical Imaging Technology, Manipal College of Health Professions, Manipal Academy of Higher Education, Manipal, Karnataka, 576104, India; 2 Department of Radio Diagnosis and Imaging, Kasturba Medical College, Manipal Academy of Higher Education, Manipal, Karnataka, 576104, India; 3 Philips Research and Development, Philips Innovation Campus, Yelahanka, Karnataka, 560064, India

**Keywords:** Low dose, Computed tomography, Deep learning image reconstruction, Iterative reconstruction technique, Image quality

## Abstract

**Background:**

The most recent advances in Computed Tomography (CT) image reconstruction technology are Deep learning image reconstruction (DLIR) algorithms. Due to drawbacks in Iterative reconstruction (IR) techniques such as negative image texture and nonlinear spatial resolutions, DLIRs are gradually replacing them. However, the potential use of DLIR in Head and Chest CT has to be examined further. Hence, the purpose of the study is to review the influence of DLIR on Radiation dose (RD), Image noise (IN), and outcomes of the studies compared with IR and FBP in Head and Chest CT examinations.

**Methods:**

We performed a detailed search in PubMed, Scopus, Web of Science, Cochrane Library, and Embase to find the articles reported using DLIR for Head and Chest CT examinations between 2017 to 2023. Data were retrieved from the short-listed studies using Preferred Reporting Items for Systematic Reviews and Meta-analysis (PRISMA) guidelines.

**Results:**

Out of 196 articles searched, 15 articles were included. A total of 1292 sample size was included. 14 articles were rated as high and 1 article as moderate quality. All studies compared DLIR to IR techniques. 5 studies compared DLIR with IR and FBP. The review showed that DLIR improved IQ, and reduced RD and IN for CT Head and Chest examinations.

**Conclusions:**

DLIR algorithm have demonstrated a noted enhancement in IQ with reduced IN for CT Head and Chest examinations at lower dose compared with IR and FBP. DLIR showed potential for enhancing patient care by reducing radiation risks and increasing diagnostic accuracy.

## Introduction

Computed tomography (CT) plays an important role in modern diagnostic radiology and assists in the identification of various complex disorders. Over the past ten years, CT scan utilization has increased significantly globally as new clinical reasons are continually identified. An estimated 375 million CT examinations are continually performed annually worldwide, with a 3-4% annual growth rate. The demands of physicians and other health care providers, as well as technology developments, have had a considerable impact on the world market for CTs. Compared to other traditional imaging modalities, CT scans offer significantly higher radiation doses (RD) despite having significant diagnostic benefits for specific patients. Adult CT scans dramatically raise cancer risk. A positive correlation between RD and cancer risks was found. ^1^^–^^5^

A recent study reported seventeen-fold variations in high-dose CT examinations among different countries. There is a four-fold variation in effective dose [ED] for Chest and abdomen examinations with less variation for CT head in adults and suggested optimization of radiation doses. ^6^ The most recommended practice in the CT sector is to reduce CT radiation exposure as low as reasonably achievable while maintaining the Image Quality (IQ). Reducing the exposure factors of tube voltage (kVp) and tube current (mA) reduces RD but increases image noise. ^7^^,^^8^ Up until ten years ago, Filtered back projection (FBP) was the only technique used for image reconstruction in CT. Although this method produces high-quality images it has noise issues at low doses and is prone to artifacts. Although an iterative reconstruction (IR) method was proposed in 1970, computational power restrictions prevented its widespread use in clinical settings. The Hybrid Iterative reconstruction (HIR) method was introduced in 2009 which had low computation time and allowed it to be implemented in clinical practice. The HIR combines iteratively reconstructed images in the raw data domain with FBP images to reduce image noise (IN). The first complete model-based iterative reconstruction (MBIR) received FDA approval in 2011. Compared to the HIR technique, this reconstruction minimizes artifacts and noise. However, it requires a greater computational power demand, which results in lengthy reconstruction times. IR techniques, irrespective of type produce lower IN, artifacts, or both at lower doses than FBP. ^9^^–^^15^ However, in general, IR at higher levels of reconstruction may result in an artificial, plastic-looking, and blotchy appearance that would eventually lower the IQ and compromise the clinician’s ability to diagnose pathologies, limiting the potential for significant RD reduction. ^16^^–^^18^

Deep learning image reconstruction algorithms (DLIR) are the most recent developments in CT image reconstruction technology. DLIRs are increasingly replacing IR techniques due to their disadvantages such as negative image texture and nonlinear spatial resolutions. DLIR is based on deep convolution neural networks (CNN) which learn from the input data sets. It gains the ability to distinguish actual signal and IN through training using pairs of low and high-quality images. In comparison to FBP and IR, the trained CNNs can distinguish between noise and signal much better, allowing for better dose reduction while preserving the image quality. DLIR produces an image texture similar to that of FBP even at low doses and high strengths. DLIR technique may be able to detect the low contrast lesions at low doses without damaging the image texture. ^19^^–^^22^ More research is required to determine the potential applications of DLIR in clinical settings. Our literature search showed there is no systematic review performed in head and chest CT examinations using Deep learning reconstruction algorithm for reducing RD and improving IQ in CT. Hence, the purpose of the article is to review the influence of DLIR on RD, IN, and outcomes of the studies compared with IR and FBP in CT Head and Chest examinations.

## Methods

### Design

This review was carried out as per the “Preferred Reporting Items for Systematic Reviews and Meta-analysis (PRISMA)” guidelines. ^23^

### Literature search strategy

A comprehensive literature search was performed using databases such as “PubMed, Scopus, Web of Science, Cochrane Library, and Embase” to find the relevant original studies ( Table 1). The MeSH terms such as “Deep Learning Image Reconstruction” “Radiation dose” “Image quality”, “Head and Chest Computed Tomography” were used ( Table 2). The search was limited to the English language including both adult and paediatric populations of Head and Chest CT examinations.

**Table 1.** Study retrieval method from database.

Database	Number of studies retrieved	Total
PubMed	32	196
Scopus	52	
Web of Science	58	
Cochrane Library	8	
Embase	46	

**Table 2.** Participants intervention comparison and outcome methodology for determining study selection criteria.

Characteristics	Criteria
Study year	2017-2023
Study Type	Cohort study
Population	Patients undergoing CT Head and Chest examinations Adult and Paediatric Population
Intervention	Image reconstruction algorithms
Comparator	DLIR vs IR or FBP
Outcomes	Radiation dose and Image quality

CT: Computed Tomography; DLIR: Deep Learning Image Reconstruction; IR: Iterative Reconstruction; FBP: Filtered Back Projection.

### Selection criteria

Articles were screened considering the Participant’s Intervention Comparison and Outcome (PICO) methodology. Case studies, case reports, conference abstracts, letters, editorial reviews, meta-analyses, or surveys were not included. The title and abstract of all the articles were independently and blindly screened by the two researchers. The articles that described a comparison of DLIR algorithms with the IR technique or FBP were included in the final review. The exclusion criteria were phantom studies, physics-based performance of DLIR, other language than English, articles with no comparison of DLIR with FBP, HIR/MBIR, and articles with no Hounsfield Unit (HU), Contrast to Noise Ratio (CNR), Signal to Noise Ratio (SNR).

### Data extraction

Data from each article was assessed independently by two researchers and any differences were solved by the third researcher.

### Quality assessment

To evaluate the quality of all the included articles, the custom-made Quality Assessment (QA) scale was used. ^24^ The list of all the questions for the quality assessment (underlying data). A score of 1 was given if the answer to the question was “yes” and each study was assigned a score ranging from 0 to 18. Based on the total scores obtained by each study, the studies were classified into three quality levels: Low- quality studies (score of 6 or lower), Moderate-quality studies (score between 7 and 11), High-quality studies (score of 12 or more).

## Results

**Figure 1.**
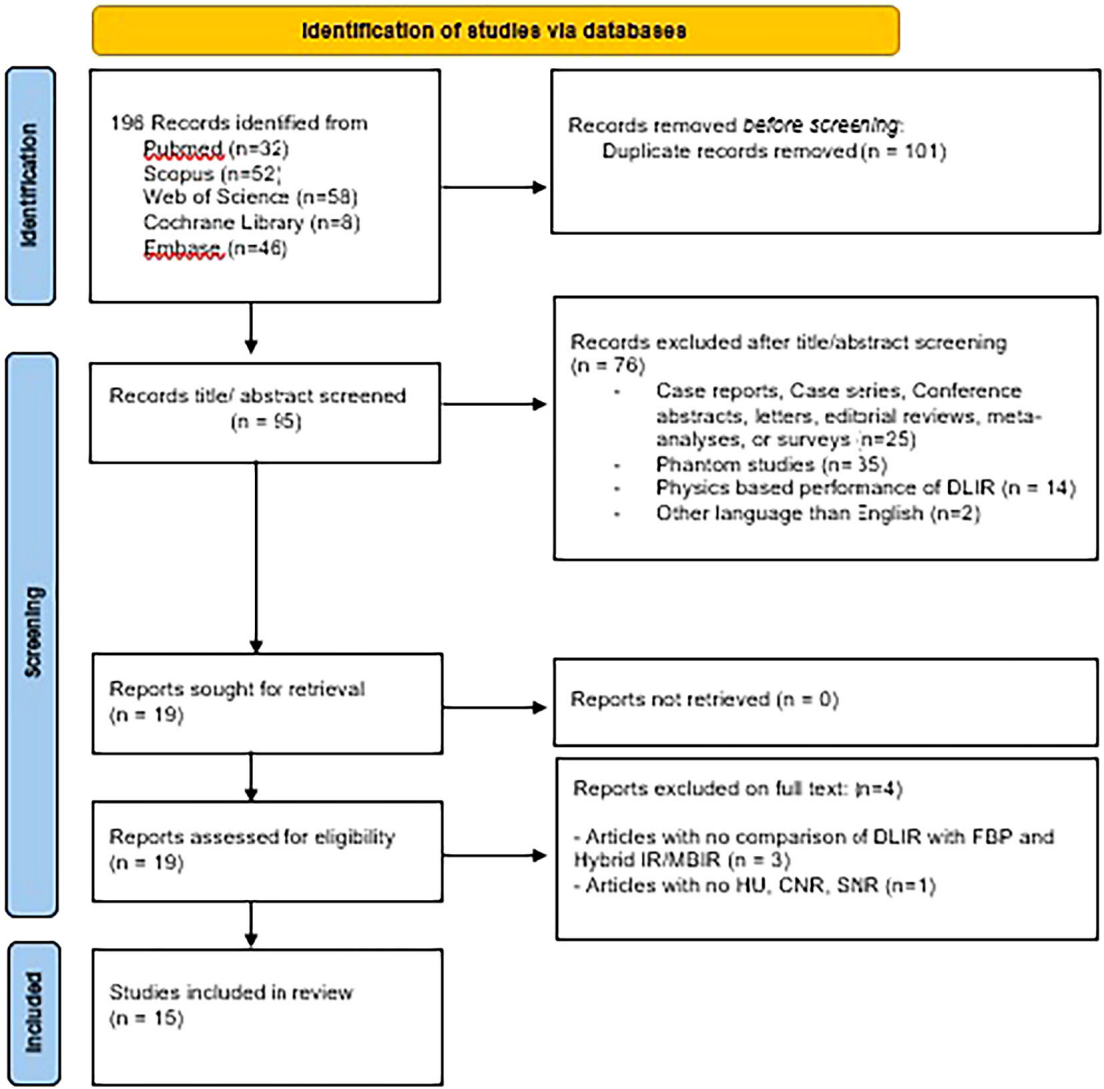
Flow chart for study selection.

Finally, 15 articles were included ( Figure 1).

### Study selection

The search in PubMed, Scopus, Web of Science, Cochrane Library and Embase resulted in 196 studies. 101 duplicates were removed. The title and abstract of 95 studies were assessed and 76 studies were excluded as they did not meet the inclusion criteria. A total of 19 reports were sought for retrieval. A total of the full text of 19 articles were assessed for eligibility. Among them, 4 articles were excluded (3 studies were excluded due to no comparison of DLIR with FBP and HIR/MBIR, and 1 article were excluded due to lack of HU, CNR, and SNR). Finally, 15 articles were included in the systematic review.

### Characteristics of selected studies

CT imaging has increased recently with the advancement in CT technology. The studies included in the review covered different countries such as China (n = 8), Japan (n = 1), France (n = 1), Korea (n = 3), Netherland (n =1), Sweden (n = 1). The RD data and IQ parameters were collected from different CT vendors such as General Electric (GE) Health care (128, 256, 512-slice, and dual-energy CT), Siemens Healthineers (256-slice), Canon Medical system (320 and 640-slice). A total sample size of 1292 was collected from the included studies. 4 studies used prospective data collection, and 11 studies used retrospective data collection. The characteristics of the study and the outcomes of each study are summarized in Table 3.

**Table 3.** Characteristics of selected studies for Head and Chest CT examinations.

Author; Year and Country	Method	CT exam	Adult/Paediatric	Reconstruction techniques	Slice CT/Vendor	Sample	QLA	QUA & Region & Lesion detection	Outcome of the study
**CT Head (Adult)**									
Alagic et al., 2022 Sweden ^25^	RS	Non-contract CT Brain	Adult	AS-V (50%), DLIR-(L-H)	512 Slice/GE	94	IN, brain structures, posterior fossa artifacts	CT, SD of GM, WM, ICH, SNR in the GM, WM and ICH Intracranial hemorrhage conspicuity	With substantially less non-diagnostic images, greater SNR (82.9%), and higher CNR (53.3%) compared to AS-V (50%), IQ of CT Brain with DLIR-M&H revealed dramatically increased IQ.
Kim I. et al., 2021 Korea ^26^	RS	Non contrast Brain	Adult	AS-V (30%), DLIR (L-H)	512- slice/GE	62	Artefacts, IQ, IN	CT HU of GM, Image Noise, Artifact index, CNR – Basal ganglia and Centrum semiovale. SNR & SD, Reconstruction time	DLIR(M&H) exhibited reduction in IN and artifacts in Posterior fossa compared with AS-V.
Nagayama et al., 2023 Japan ^27^	RS	Non-Contrast CT Brain	Adult	LD-HIR, MBIR, DLIR	320-Slice/CMS	114	Noise magnitude, IT, GM-WM differentiation, Artifact, IS, OIQ,	GM-WM differentiation, CNR, HU, SD GM, WM Lesion Conspicuity	DLIR can enhance the IQ of the CT Brain while reducing low RD and reconstruction time.
Oostveen et al., 2021 Netherland ^28^	RS	Non-Contrast CT Brain	Adult	DLIR, MBIR, HIR	320-slice/CMS	50	IN, IS, natural appearance, GWM diff., artefacts, OIQ	SNR & SD of CSF, Reconstruction time	Comparing DILR to MBIR and HIR leads in reduced IN and better tissue differentiation with a little increase in reconstruction time.
**CT Head (Pediatric)**									
Sun J et al., 2021China ^29^	RS	Non contrast CT Brain	Pediatric	FBP, AS-V 50% and 100% DLIR (High)	256-slice/GE	50	Clarity of cistern boundaries, GWM differentiation, Image quality	GM HU and SD, WM HU and SD, GM SNR, WM SNR,CNR	In comparison to AS-V and FBP, DLIR-H demonstrated reduced IN and increased IQ. Lesion identification is improved in 0.625 mm DLIR-H images, which also have similar image noise levels to 5 mm AS-V (50%) images.
**CT Chest (Adult)**									
Ferri et al., 2022 France ^30^	RS	Non-contrast chest CT	Adult	FBP, AS-V 70% and DLIR (L-H)	256-Slice/GE	54	OIQ,IN, artefacts	HU, SD, SNR (Air, trachea, muscle). Emphysema volume	SNR was significantly raised with DLIR. Emphysema volume decrease with increase strengths of DLIR.
Jiang B et al., 2022 China ^31^	PS	Non contrast and contrast Chest	Adult	AS-V (40%, 80% and DLIR- M, H.	256-slice/GE	203	Lung tissue noise, air background noise Nodule detection rate Malignant-related features	Measurement accuracy of nodule detection.	DLIR showed better enhancement of nodule detection rate and reduced image noise compared with AS-V images. For DLIR-H, 81.5% malignancy-related characteristics detections were noted.
Jiang J. M. et al., 2022 China ^32^	RS	Non contrast Chest CT	Adult	AS-V (50%) and DLIR (L-H)	256-slice/GE	50	Soft tissue and lung tissue	SD, HU of (Aorta, Lung, Muscle, Liver, Vertebrae), CNR, SNR.	At the same dosage, DLIR can deliver a greater IQ, boosting the doctors DC and raising the diagnostic accuracy of LDCT.
Jo et al., 2023 korea ^33^	RS	Non contrast Chest CT	Adult	ADMIRE and DLIR (L-H)	Dual Source/Somatom Force	100	SN, Spatial resolution, Distortion artifact, Beam hardening artifact, OIQ	Noise, SNR, Edge rise distance Nodule detectability assessment	When compared to normal LDCT images reconstructed using IR, Quarter LD images reconstructed with DLIR demonstrated noninferior nodule detectability and IQ.
Kim J. H. et al., 2021 Korea ^34^	RS	Non-Contrast Chest CT	Adult	AS-V 30% and (DLIR M & H)	512 Slice/GE	58	IC, IN & conspicuity of structures	HU, SD, SNR and CNR (Lung, Mediastinum, Liver Air)	Compared with AS-V 30% the DLIR images exhibited IN reduction in LDCT images by maintaining IQ.
Kim C. H. et al., 2022 China ^35^	RS	Non contrast chest CT	Adult	FBP, ASiR-V 30%, DLIR	512 Slice/GE	193	Image contrast, OIQ	HU, SD, SNR and BRISQUE Score of lower lobes Diagnostic characterization of usual interstitial pneumonia (UIP)	DLIR images produced highest OIQ score. Comparing DLIR to AS-V and FBP, IN, SNR, and visual rating of chest LD-CT scan images all improved. For purpose of diagnosing UIP DLIR may be useful.
Tian et al., 2022 China ^36^	RS	Non contrast Chest CT	Adult	AS-V 40%, DLIR (L-H)	256-slice/GE	86	IN, Image artifacts, and lesions.	HU, SD, (Lung Muscle, Fat, Aorta)	Compared with AS-V 40%, DLIR effectively reduced IN and improved IQ in LD chest CT.
Wang et al., H 2022 China ^37^	PS	Non contrast chest CT	Adult	AS- V 40%, DLIR M and H	256-slice/GE	48	Nodules, Lung tissue, Artifacts and diagnostic confidence	Objective Noise, CNR, Image Signal (Aorta)	With DLIR, LD chest CT scans minimise IN, while DLIR-H provides images with a similar level of quality to SDCT AS (40%) while using just 4% of the RD.
Wang J et al., 2023 China ^38^	PS	Non contrast Chest CT	Adult	HIR, DLIR	640-slice/CMS	60	OIQ	SD, HU, and SNR (Aorta, Paraspinal muscle,fat) Assessment of Pulmonary lesion Conspicuity	In comparison to HIR, LDCT-DLIR offers an excellent IQ with the exception of sub-solid nodules and reduced lung attenuation.
Zhao et al., 2022 China ^39^	PS	HRCT and Low dose Chest	Adult	HIR (AIDR3D, LDCT with DLIR	320-slice/CMS	70	OIQ, SIN, artifacts	SNR Lung in ILD	Low dose CT DLIR showed better recognition of ground glass opacity and visualization of architectural distortion than HRCT-AIDR

AIDR: Adaptive iterative dose reduction method 3D; AS-V: Adaptive statistical iterative reconstruction-V; CMS: Cannon medical systems; CT: Computed Tomography; CNR: Contrast to Noise ratio; DC: Diagnostic confidence; DLIR: Deep learning image reconstruction; GM: Gray matter; HU: Hounsfield Unit; HIR: Hybrid iterative reconstruction; H: High; HRCT: High resolution computed tomography;IN: Image noise; IQ: Image quality; IC: Image contrast; IT: Image texture; IS: Image sharpness; ICH: Intracranial hemorrhage; ILD: Interstitial lung disease; LD: Low dose; M: Medium; L: Low; NT: Noise texture; MBIR: Model based iterative reconstruction; OIQ: Objective image quality; PS: Prospective study; QLA: Qualitative analysis; QUA: Quantitative analysis; RD: Radiation dose; RS: Retrospective study; SD: Standard dose; sd: Standard deviation; SR: Spatial resolution; SNR: Signal to Noise ratio; SIN: Subjective image noise; UHRCT: Ultrahigh resolution CT; WM: White matter.

### Quality assessment

The results of the quality assessment are summarized in Table 4. All studies compared DLR to hybrid iterative reconstruction techniques. 5 studies compared DLIR with IR and FBP algorithms. A total of 14 studies were rated as high and 1 study as moderate quality.

**Table 4.** Quality scores of the selected studies.

	CT Head	Alagic et al. ^25^ 2022	Kim I et al. ^26^ 2021	Nagayama et al. ^27^ 2023	Oostveen et al. ^28^2021	Sun J et al. ^29^ 2021	CT Thorax	Ferri et al. ^30^ 2022	Jiang B et al. ^31^ 2022	Jiang J M et al. ^23^ 2022	Jo et al. ^33^ 2023	Kim J. H et al. 2021	Kim C. H et al. ^34^ 2023	Tian et al. ^36^ 2022	Wang H et al. ^37^ 2022	Wang J et al. ^38^ 2023	Zhao et al. ^39^ 2022
1.		0	0	0	0	0		0	1	0	0	0	0	0	1	1	1
2.		1	1	1	1	1		1	1	1	1	1	1	1	1	1	1
3i.		1	1	1	1	1		1	1	1	1	1	1	1	1	1	1
3ii.		1	1	1	1	1		1	1	1	1	1	1	1	1	1	1
3iii.		1	1	1	1	1		1	1	1	1	1	1	1	1	1	1
3iv.		1	1	1	1	1		1	1	1	1	1	1	1	1	1	1
3v.		1	1	1	0	0		0	1	0	1	0	1	1	0	0	1
3vi.		0	0	1	0	0		0	1	0	0	0	1	0	0	0	0
4i.		1	1	1	1	1		1	1	1	1	1	1	1	1	1	1
4ii.		1	1	0	0	1		0	1	0	1	0	0	0	0	0	0
4iii.		1	1	1	1	1		1	1	1	1	1	1	1	1	1	1
5.		1	1	1	1	1		0	0	1	1	0	0	1	0	1	0
6i.		1	1	1	1	1		1	1	1	1	1	1	1	1	1	1
6ii.		1	1	1	1	1		1	1	1	1	1	1	1	1	1	1
7i.		1	1	1	1	1		1	1	1	1	1	1	1	1	1	1
7ii.		0	0	0	0	1		1	1	0	0	0	1	0	1	0	0
8i.		0	0	0	1	0		0	0	0	0	0	0	0	0	0	0
8ii.		1	1	1	1	0		1	1	1	1	1	1	1	1	1	1
Total		14	14	14	13	13		12	16	12	14	11	14	13	13	13	13

### Image noise (IN) and radiation dose (RD) reduction in CT head and chest examinations

***CT head examination**:* We summarized the percentage IN and RD reduction for various CT examinations in Table 5. A total of five studies in CT brain that used DLIR showed a reduction in IN (18-52%) compared with IR and FBP. ^25^^–^^29^ In the brain, a study compared SD with IR (CTDIvol-70.8 mGy & EF-2.8±0.2 mSv) and LD with DLIR (CTDIvol-53.0 mGy & ED-2.1±0.1 mSv) protocol in adult CT brain and noticed a 25% reduction in RD. ^27^ Another study was done with LD DLIR (CTDIvol-18.18 mGy, DLP- 269.43 mGy.cm) protocol of CT brain. ^29^

**Table 5.** Radiation dose parameters and percentage reduction in radiation dose, image noise of CT Head and Chest.

S.No	Author	CTDI vol (mGy)	DLP (mGy.cm)	SSDE (mGy)	ED (mSv)	% reduction in radiation dose	% reduction in Image noise
**CT Head (Adult)**							
1.	Alagic et al 2022 ^25^	Mean 46.96±0.49	Mean 847.84±22.25	-	-	-	DLIR H reduced IN by 37.2% (AS- V 50)
2.	Kim et al 2021 ^26^	35.90±3.36	768.38±86.61	-	-	-	DLIR H reduced 52.25 compared to AS V 30%
3.	Nagayama et al 2023 ^27^	SD-70.8 LD-53.0	-	-	SD-2.8±0.2 LD-2.1±0.1	25%	LD DLIR reduced IN to 22.2% compared to LD HIR
4.	Oostveen et al 2021 ^28^	16.1-52.7	-	-	-	-	DLIR showed reduce IN to 23.31 compared to MBIR and 11% compared to HIR
**CT Head (Pediatric)**							
5.	Sun J et al 2021 ^29^	LD-18.18±2.82	LD-269.43±57.95	-	-	-	DLIR-H showed reduced IN to 5.5% compared to AS-V 100% and 18% compared to AS-V 50%
**CT Chest (Adult)**							
6.	Ferri et al 2022 ^30^	LD-2.38±0.68	LD-98.7±26.5	-	-	-	DLIR H reduced IN by 35.1% compared with AS-V
7.	Jiang B et al 2022 ^31^	CECT-4.9±0.7 ULD1-0.13 ULD2-0.27	CECT-169.9±26.4 ULD1-5.1±0.3 ULD2-10.2±0.6	-	CECT-2.38±0.37 ULD1-0.07 ULD2-0.14	94-97%	DLIR H reduced image noise by 9% compared to AV 40%
8.	Jiang J M et al 2022 ^32^	LD-2.04	LD-79.69±4.81	LD-1.07±0.07	-	-	DLIRH reduced IN by 11%.5 compared with AS V
9.	Jo et al 2023 ^33^	QLD-0.29 LD-1.2	QLD-11.6 LD-46.4	-	QLD-0.16 LD-0.65	75%	QLD-DLIR showed reduced IN by 28.1 compared with ADMIRE
10.	Kim et al 2021 ^34^	LD-1.07	LD-53.9±2.3	LD-0.69±0.05	LD-0.75±0.03	-	DLIR H reduced IN to 18.33% compared with AS V 30%
11.	Kim et al 2023 ^35^	LD-1.96±0.03	LD-70.32±5.82	-	LD-0.98±0.08	-	DLIR M reduced IN to 14% compared with AS V 30%
12.	Tian et al (2022) ^36^	-	-	-	LD-1.03±0.36	-	DLIR H reduced IN to 33.3% compared to AS-V 40%
13.	Wang H et al 2022 ^37^	SD-12.46±1.16 LD-0.54	SD-447.32±34.51 LD-19.44±1.37	-	-	95%	DLIR-H reduced IN to 56.29% compared to AS V 40%
14.	Wang J et al 2023 ^38^	SD-4.88±1.56 (3.10-9.90) LD-0.70	SD-146.08±46.49 (77.17-311.94) LD-20.54±2.10 (14.29-23.84)	-	SD-2.05±0.65 (1.08-4.37) LD-0.29±0.03 (0.20-0.33)	85%	LDCT DLIR H reduced IN to 33.8% compared to LDCT HIR
15.	Zhao et al 2022 ^39^	HRCT-5.38±1.49 LDCT-2.00	228.99±62.69 (HRCT) 87.38±6.01 (LDCT)	7.29±1.45 (HRCT) 2.78±0.26 (LDCT)	1.93±0.55 (HRCT) 0.72±0.07	61.9%	DLIR H showed reduced IN to 30.76% compared to AIDR

AIDR: Adaptive iterative dose reduction method 3D; AS-V: Adaptive statistical iterative reconstruction-V; CECT: Contrast Enhanced CT; DLIR: Deep learning image reconstruction; DLP; Dose length product; ED: Effective dose; FIRST: Forward projected model-based iterative reconstruction solution; H- High, HIR: Hybrid iterative reconstruction; HRCT: High resolution computed tomography; IN:Image noise; kVp: kilo voltage; QLD: Quarter Low dose; LD: Low dose; M: Medium; ULD: Ultra low dose; SSDE: Size specific dose estimate; SD: Standard dose.

***CT chest examination:*** A total of nine studies from chest CT that used DLIR showed a reduction in IN (9-50%) compared with IR and FBP. ^30^^–^^39^ Four studies compared LD with DLIR and SD with IR for chest CT and observed a reduction (62-97%) in RD. ^31^^,^^37^^–^^39^ Another 4 studies done with LD chest CT with DLIR [CTDI vol (1.07-2.38 mGy.cm), DLP (79.69-08.7 mGy.cm), SSDE (0.69-1.07 mGy), ED (0.98-1.03 mSv)]. ^30^^,^^32^^,^^34^^,^^35^ One study done with Quarter low dose (QLD) CT Chest using DLIR showed 75% reduction in radiation dose compared to IR. ^33^

## Discussion

This systematic review focussed on investigating the influence of DLIR on RD, IN, and outcomes of the studies compared with IR and FBP in Head and Chest CT examinations.

### CT head

Our review noted that for CT Brain examination, DLIR (Medium and High) showed reduced IN (18-52%), improved IQ (GM-WM differentiation) with better detection of cerebral lesions, and reduced RD (25%). In the Pediatric CT brain, a study by Sun et al. noted that higher strength DLIR reduced image noise and noted better detection of cerebral lesions in 0.625 mm compared to 5 mm slice thickness. The thinner sections of DLIR-H were able to identify micro-hemorrhages of less than 3 mm. ^29^ Nagayama et al. demonstrated a 25% reduction in RD with LD CT-DLIR (120 kVp, 280 mA) compared to SD -IR (120 kVp, 350 mA) and also observed that DLIR had the highest sensitivity in lesion detection (2.9±0.2) compared to MBIR (1.9±0.5) and HIR (1.2±0.4) in adult CT brain. ^27^ Studies by Oostveen et al. and Nagayama et al. showed reduced reconstruction times 44 sec; 24±1 sec compared to MBIR (176 s & 319±17 secs) for Non-contrast CT brain. ^27^^–^^28^ Studies by Alagic et al. and Sun et al. reported CTDIvol and DLP of 46.96±0.49 mGy; 847.84±2.25 mGy.cm and 18.18±2.82 mGy; 269.3±57.95 mGy.cm in adult and pediatric CT Head respectively. ^25^^,^^29^

### CT chest

Our review noted that LDCT of the chest with DLIR showed higher image contrast and lower IN (9-56%) and reduced RD (62-97%) compared with FBP and IR. Zhao et al. compared LDCT with DLIR (120kvp, 30 mAs) and HRCT with HIR (120kVp, Automatic tube current modulation) for the patients with interstitial lung disease (ILD) and noted that LDCT DLIR showed better visualization of honeycombing and assessment of bronchiectasis. ^39^ Kim et al. noted that DLIR-H yielded higher scores in determining the prominence of the lungs main structures of the lungs. ^34^ Jiang et al. noted that ULD-CT with DLIR under or overestimated the long diameter and sub-solid nodules compared with CECT Thorax. DLIR-H overestimated the solid and calcified nodules while underestimating the long diameter and amount of sub-nodules. ^31^ Wang et al. noted LDCT with DLIR provides higher scores for assessing pulmonary lesions except for sub-solid nodules or ground glass opacity nodules (GGN) compared to SD with HIR, whereas GGN greater than 4 mm can be picked up on LDCT DLIR images. ^38^ Tian et al. reported that DLIR-H appeared to be slightly smoothed and DLIR M provides higher structures on visualization of smoother structures. ^36^ Ferri et al. reported DLIR reconstruction series provided the smallest volume of emphysema compared with Adaptive statistical iterative reconstruction-V (ASIR-V) and FBP and also observed the increase in strength of DLIR led to a decrease in the size of emphysema. ^30^

The study has a few limitations. Firstly, we did not include phantom studies. Secondly, we did not perform meta-analysis due to heterogeneity in terms of scanners and protocols used for head and chest examinations. The adoption of DLIR algorithms holds promise for improving IQ, reducing RD, and mitigating IN in Head and Chest CT examinations compared to traditional IR and FBP techniques. Healthcare providers may consider incorporating DLIR into their imaging protocols to enhance patient care by reducing radiation risks while maintaining diagnostic accuracy. Furthermore, future research efforts should focus on optimizing DLIR algorithms, investigating their long-term effects on patient outcomes, and evaluating cost-effectiveness compared to conventional reconstruction methods. Additional studies exploring the application of DLIR in other anatomical regions and patient populations could further expand its utility and impact on healthcare delivery.

## Conclusion

In conclusion, DLIR is a versatile and valuable technology that consistently improves IQ, enhances lesion detection, reduces radiation exposure, and mitigates image artifacts across a wide range of medical imaging applications compared with IR and FBP. A careful selection of strengths of DLIR, slice thickness and radiation dose levels are required for evaluation of tiny lesions, which can overcome with next generation DLIR algorithms. Overall, DLIR holds promise for improving patient care and diagnostic accuracy in various clinical settings.

### Ethics and consent

The study did not involve any human participants and only systematic review was conducted; hence the written informed consent and Institutional ethical committee approval (IEC) was not required.

## Data Availability

No data is associated with this article. Figshare: F1000 DLIR Systematic Review. https://doi.org/10.6084/m9.figshare.25404226.v3. ^40^ This project contains the following underlying data:
• Quality assessment scale • PRISMA Chart Figshare: PRISMA_2020_checklist.pdf, https://doi.org/10.6084/m9.figshare.25404226.v3. ^40^ Data are available under the terms of the Creative Commons Attribution 4.0 International license (CC-BY 4.0)

## References

[1] Smith-Bindman R Miglioretti DL Johnson E : Use of Diagnostic Imaging Studies and Associated Radiation Exposure for Patients Enrolled in Large Integrated Health Care Systems, 1996-2010. *JAMA.* 2012;307(22):2400–2409. 10.1001/jama.2012.5960 22692172 PMC3859870

[2] Pola A Corbella D Righini A : Computed tomography use in a large Italian region: trend analysis 2004-2014 of emergency and outpatient CT examinations in children and adults. *Eur. Radiol.* 2018;28(6):2308–2318. 10.1007/s00330-017-5225-x 29318431

[3] Mettler FA Huda W Yoshizumi TT : Effective Doses in Radiology and Diagnostic Nuclear Medicine: A Catalog1. *Radiology.* 2008;248(1):254–263. 10.1148/radiol.2481071451 18566177

[4] Agostini A Borgheresi A Granata V : Technological advances in body CT: a primer for beginners. *Eur. Rev. Med. Pharmacol. Sci.* 2022;26(21):7918–7937. 10.26355/eurrev_202211_30144 36394741

[5] Cao CF Ma KL Shan H : CT Scans and Cancer Risks: A Systematic Review and Dose-response Meta-analysis. *BMC Cancer.* 2022;22(1):1238. 10.1186/s12885-022-10310-2 36451138 PMC9710150

[6] Smith-Bindman R Wang Y Chu P : International variation in radiation dose for computed tomography examinations: prospective cohort study. *BMJ.* 2019;364:k4931. 10.1136/bmj.k4931 30602590 PMC6314083

[7] Baskan O Erol C Ozbek H : Effect of radiation dose reduction on image quality in adult head CT with noise-suppressing reconstruction system with a 256 slice MDCT. *J. Appl. Clin. Med. Phys.* 2015;16(3):285–296. 10.1120/jacmp.v16i3.5360 26103494 PMC5690139

[8] Kubo T Ohno Y Kauczor HU : Radiation dose reduction in chest CT-Review of available options. *Eur. J. Radiol.* 2014;83(10):1953–1961. 10.1016/j.ejrad.2014.06.033 25066756

[9] Willemink MJ Noël PB : The evolution of image reconstruction for CT-from filtered back projection to artificial intelligence. *Eur. Radiol.* 2019;29(5):2185–2195. 10.1007/s00330-018-5810-7 30377791 PMC6443602

[10] Willemink MJ De Jong PA Leiner T : Iterative reconstruction techniques for computed tomography Part 1: Technical principles. *Eur. Radiol.* 2013;23:1623–1631. 10.1007/s00330-012-2765-y 23314600

[11] Willemink MJ Leiner T De Jong PA : Iterative reconstruction techniques for computed tomography part 2: initial results in dose reduction and image quality. *Eur. Radiol.* 2013;23:1632–1642. 10.1007/s00330-012-2764-z 23322411

[12] Hara AK Paden RG Silva AC : Iterative reconstruction technique for reducing body radiation dose at CT: Feasibility study. *Am. J. Roentgenol.* 2009;193(3):764–771. 10.2214/AJR.09.2397 19696291

[13] Geyer LL Schoepf UJ Meinel FG : State of the Art: Iterative CT Reconstruction Techniques. *Radiology.* 2015;276(2):339–357. 10.1148/radiol.2015132766 26203706

[14] Han WK Na JC Park SY : Low-dose CT angiography using ASiR-V for potential living renal donors: a prospective analysis of image quality and diagnostic accuracy. *Eur. Radiol.* 2020;30(2):798–805. 10.1007/s00330-019-06423-1 31471753

[15] Moloney F James K Twomey M : Low-dose CT imaging of the acute abdomen using model-based iterative reconstruction: a prospective study. *Emerg. Radiol.* 2019;26(2):169–177. 10.1007/s10140-018-1658-z 30448900

[16] Stiller W : Basics of iterative reconstruction methods in computed tomography: A vendor-independent overview. *Eur. J. Radiol.* 2018;109:147–154. 10.1016/j.ejrad.2018.10.025 30527298

[17] Saiprasad G Filliben J Peskin A : Evaluation of Low-Contrast Detectability of Iterative Reconstruction across Multiple Institutions, CT Scanner Manufacturers, and Radiation Exposure Levels. *Radiology.* 2015;277(1):124–133. 10.1148/radiol.2015141260 25989480

[18] Solomon J Mileto A Ramirez-Giraldo JC : Diagnostic Performance of an Advanced Modeled Iterative Reconstruction Algorithm for Low-Contrast Detectability with a Third-Generation Dual-Source Multidetector CT Scanner: Potential for Radiation Dose Reduction in a Multireader Study. *Radiology.* 2015;275(3):735–745. 10.1148/radiol.15142005 25751228

[19] Zhang Z Seeram E : The use of artificial intelligence in computed tomography image reconstruction - A literature review. *J. Med. Imaging Radiat. Sci.* 2020;51(4):671–677. 10.1016/j.jmir.2020.09.001 32981888

[20] Koetzier LR Mastrodicasa D Szczykutowicz TP : Deep Learning Image Reconstruction for CT: Technical Principles and Clinical Prospects. *Radiology.* 2023;306(3):e221257. 10.1148/radiol.221257 36719287 PMC9968777

[21] Timothy P Szczykutowicz GVT Dhanantwari A : A Review of Deep Learning CT Reconstruction: Concepts, Limitations, and Promise in Clinical Practice. *Curr. Radiol. Rep.* 2022;10:101–115.

[22] Jensen CT Liu X Tamm EP : Image quality assessment of abdominal CT by use of new deep learning image reconstruction: Initial experience. *Am. J. Roentgenol.* 2020;215(1):50–57. 10.2214/AJR.19.22332 32286872

[23] Page MJ Mckenzie JE Bossuyt PM : The PRISMA 2020 statement: an updated guideline for reporting systematic reviews. *BMJ.* 2021;372:n71. 10.1136/bmj.n71 33782057 PMC8005924

[24] Abel van Stiphout J Driessen J Koetzier LR : The effect of deep learning reconstruction on abdominal CT densitometry and image quality: a systematic review and meta-analysis. *Eur. Radiol.* 2022;32:2921–2929. 10.1007/s00330-021-08438-z 34913104 PMC9038933

[25] Alagic Z Diaz Cardenas J Halldorsson K : Deep learning versus iterative image reconstruction algorithm for head CT in trauma. *Emerg. Radiol.* 2022;29:339–352. 10.1007/s10140-021-02012-2 34984574 PMC8917108

[26] Kim I Kang H Yoon HJ : Deep learning-based image reconstruction for brain CT: improved image quality compared with adaptive statistical iterative reconstruction-Veo (ASIR-V). *Neuroradiology.* 2021;63(6):905–912. 10.1007/s00234-020-02574-x 33037503

[27] Nagayama Y Iwashita K Maruyama N : Deep learning-based reconstruction can improve the image quality of low radiation dose head CT. *Eur. Radiol.* 2023;33:3253–3265. 10.1007/s00330-023-09559-3 36973431

[28] Oostveen LJ Meijer FJA Lange F de : Deep learning–based reconstruction may improve non-contrast cerebral CT imaging compared to other current reconstruction algorithms. *Eur. Radiol.* 2021;31(8):5498–5506. 10.1007/s00330-020-07668-x 33693996 PMC8270865

[29] Sun J Li H Wang B : Application of a deep learning image reconstruction (DLIR) algorithm in head CT imaging for children to improve image quality and lesion detection. *BMC Med. Imaging.* 2021;21(1):1–9. 34238229 10.1186/s12880-021-00637-w PMC8268450

[30] Ferri F Bouzerar R Auquier M : Pulmonary emphysema quantification at low dose chest CT using Deep Learning image reconstruction. *Eur. J. Radiol.* 2022;152:110338. 10.1016/j.ejrad.2022.110338 35533559

[31] Jiang B Li N Shi X : Deep Learning Reconstruction Shows Better Lung Nodule Detection for Ultra–Low-Dose Chest CT. *Radiology.* 2022;303(1):202–212. 10.1148/radiol.210551 35040674

[32] Jiang JM Miao L Liang X : The Value of Deep Learning Image Reconstruction in Improving the Quality of Low-Dose Chest CT Images. *Diagnostics (Basel).* 2022;12(10):2560. 10.3390/diagnostics12102560 36292249 PMC9601258

[33] Jo GD Ahn C Hong JH : 75% radiation dose reduction using deep learning reconstruction on low-dose chest CT. *BMC Med. Imaging.* 2023 Sep 11;23(1):121. 10.1186/s12880-023-01081-8 37697262 PMC10494344

[34] Kim JH Yoon HJ Lee E : Validation of deep-learning image reconstruction for low-dose chest computed tomography scan: Emphasis on image quality and noise. *Korean J. Radiol.* 2021;22(1):131–138. 10.3348/kjr.2020.0116 32729277 PMC7772377

[35] Kim CH Chung MJ Cha YK : The impact of deep learning reconstruction in low dose computed tomography on the evaluation of interstitial lung disease. *PLoS One.* 2023;18:e0291745. 10.1371/journal.pone.0291745 37756357 PMC10529569

[36] Tian Q Li X Li J : Image quality improvement in low-dose chest CT with deep learning image reconstruction. *J. Appl. Clin. Med. Phys.* 2022;23(12):e13796. 10.1002/acm2.13796 36210060 PMC9797160

[37] Wang H Li LL Shang J : Application of deep learning image reconstruction in low-dose chest CT scan. *Br. J. Radiol.* 2022;95(1133). 10.1259/bjr.20210380 35084210 PMC10993973

[38] Wang J Sui X Zhao R : Value of deep learning reconstruction of chest low-dose CT for image quality improvement and lung parenchyma assessment on lung window. *Eur. Radiol.* 2023;34:1053–1064. 10.1007/s00330-023-10087-3 37581663

[39] Zhao R Sui X Qin R : Can deep learning improve image quality of low-dose CT: a prospective study in interstitial lung disease. *Eur. Radiol.* 2022;32(12):8140–8151. 10.1007/s00330-022-08870-9 35748899

[40] Kadavigere R : F1000 DLIR systematic review.[Dataset]. *figshare.* 2024. 10.6084/m9.figshare.25404226.v3

