# Supplementary material for: Influence of deep learning image reconstruction algorithm for reducing radiation dose and image noise compared to iterative reconstruction and filtered back projection for head and chest computed tomography examinations: a systematic review
Source: F1000Res. 2024 Apr 15;13:274. [Version 1] doi: 10.12688/f1000research.147345.1 (PMC11079581; doi:10.12688/f1000research.147345.1)
Supplement: Peer review history

# Reviewer report: Reviewer response for version 1

**DOI:** 10.5256/f1000research.161532.r268959

**Published:** 2024-05-29

**Authors:** Senthil Manikandan Palaniyappan

**Affiliations:** 1 Assistant professor, Department of Radiation Physics, Kidwai Memorial Institute of Oncology, Bangalore, India

**Major comments:**

 The objective of this study is to evaluate the impact of the Deep learning image reconstruction (DLIR's) algorithm on Radiation Dose (RD), Image Noise (IN), and Image Quality (IQ) in Head and Chest CT examinations. This study addresses a crucial area of research in image reconstruction technology in CT. The comprehensive approach ensures a thorough understanding of DLIR’s impact on various aspects of CT Head and Chest imaging.

 The study's methodology is well-structured and follows the PRISMA guidelines, ensuring a systematic and comprehensive approach to data collection and analysis. The inclusion of major databases ensures a comprehensive retrieval of relevant articles, enhancing the study’s credibility and readability.

 Findings regarding CT head examinations, including the reduction in image noise, improved Image quality with better lesion detection, and reduced radiation dose with DLIR compared to IR and FBP, are well documented and supported by specific studies. Similarly, the observations related to CT chest examinations, such as higher image contrast, lower image noise, and substantial reduction in radiation dose with DLIR, are clearly stated and backed by relevant research. Results mentioned in the study such as a reduction in RD 25% in adult CT brain examinations and 62% to 97% in chest CT examinations add quantitative support to the significant impact of DLIR reducing radiation exposure while maintaining diagnostic accuracy.

 Acknowledging the limitations of the study, such as the exclusion of phantom studies and heterogeneity provides transparency and enhances the credibility of acknowledging potential biases or gaps in the research process.

**Minor comments:**

 Ensure that all abbreviations are clearly defined upon first use.

**Suggestions to editor**

 The review article adds significant knowledge to the literature about the potential advantages of DLIR techniques to enhance diagnostic accuracy, minimize radiation exposure, and improve image quality in CT brain and Chest examinations. The review provides valuable insights into the advancements of DLIR in CT imaging, its benefits in reducing radiation dose and image noise while improving Image quality, and highlights the potential for further research and application of DLIR in clinical settings.

Are the rationale for, and objectives of, the Systematic Review clearly stated?

Yes

Is the statistical analysis and its interpretation appropriate?

Yes

If this is a Living Systematic Review, is the ‘living’ method appropriate and is the search schedule clearly defined and justified? (‘Living Systematic Review’ or a variation of this term should be included in the title.)

Not applicable

Are sufficient details of the methods and analysis provided to allow replication by others?

Yes

Are the conclusions drawn adequately supported by the results presented in the review?

Yes

Reviewer Expertise:

Radiation Imaging and Dosimetry

I confirm that I have read this submission and believe that I have an appropriate level of expertise to confirm that it is of an acceptable scientific standard.

# Reviewer report: Reviewer response for version 1

**DOI:** 10.5256/f1000research.161532.r268965

**Published:** 2024-05-15

**Authors:** Thayalan Kuppusamy

**Affiliations:** 1 Dr Kamakshi Memorial Hospital, Chennai, Tamil Nadu, India

Questions/Comments

 1.What is the estimated CT examinations number in India, including pediatric?

 2. Why abdomen CT is not taken for the study since it involves larger dose variation .

 3.Why phantom study is excluded,  it is basic method and gold standard of dose estimation?

 4.Image noise (IN) depends on type of noise filters used- will all 15 articles used had  same noise filter?

 5. Is slice thickness is considered as a parameter in deciding radiation dose?

 6.The RD data and IQ parameter collected from various CT vendors shows variation, means not homogeneous? Comparing the RD with them will result a meaningful data  or not?

 Why Phillips vendor is not included since large number of installation is here in India

 7.CT radiation dose and cancer risk is debatable, it can not be taken as  hypothesis.  Probability of  cancer  induction is less if the dose is < 100 mSv. No CT scan offer such magnitude of radiation now  a days.

 8.There was no mention about diagnostic reference level (DRL), especially in India.  Is the stated DLIR  brings the dose below the DRL or not ?

 9. CDDI, focal spot, slice thickness, filtration (bow type filter), tube rotation time, pitch, current modulation, AEC, low dose protocol, kV, mAs, scan area  may differ in each CT and influence dose- all the parameter are accounted?

Are the rationale for, and objectives of, the Systematic Review clearly stated?

Yes

Is the statistical analysis and its interpretation appropriate?

Yes

If this is a Living Systematic Review, is the ‘living’ method appropriate and is the search schedule clearly defined and justified? (‘Living Systematic Review’ or a variation of this term should be included in the title.)

Yes

Are sufficient details of the methods and analysis provided to allow replication by others?

Partly

Are the conclusions drawn adequately supported by the results presented in the review?

Yes

Reviewer Expertise:

Radiation physics , diagnostic radiology

I confirm that I have read this submission and believe that I have an appropriate level of expertise to confirm that it is of an acceptable scientific standard.

# Reviewer report: Reviewer response for version 1

**DOI:** 10.5256/f1000research.161532.r268955

**Published:** 2024-05-08

**Authors:** Dr. Tamijeselvan S

**Affiliations:** 1 College of MRIT, Mother Teresa Postgraduate and Research institute of Health sciences, Indira Nagar, Puducherry, India

**Major comments**

 The review article provides comprehensive review of the  Deep Learning Image Reconstruction (DLIR)  and its application on radiation dose and image noise. This review particularly concentrating on the application  and outcomes in head and chest Computed Tomography examinations compared to traditional Iterative (IR) and Filtered Back Projection (FBP) techniques. In the methodology section the authors follow the Preferred Reporting Items for Systematic Reviews and Meta-Analyses (PRISMA), which is an evidence-based minimum set of items for reporting in systematic reviews and meta-analyses. It has been clearly explained.

 In the results the author clearly explains the key outcome in related to the aim and objectives. In particular DLIR's influence on radiation dose reduction, image noise reduction, and enhancement of image quality in CT head and chest examinations were clearly explained. The discussion and conclusion effectively explain the results,

**Minor comments**

 Some more references may be added to support the review article. The article may include the data of various other anatomical part like thorax.

**Suggestions to editor:**

 On a whole , it is an excellent review article that represents a valuable contribution for the dose reduction in the  CT scan using proper image reconstruction technology. Also this article provides the impact of Deep Learning Image Reconstruction technology on improvement of image quality.

Are the rationale for, and objectives of, the Systematic Review clearly stated?

Yes

Is the statistical analysis and its interpretation appropriate?

Yes

If this is a Living Systematic Review, is the ‘living’ method appropriate and is the search schedule clearly defined and justified? (‘Living Systematic Review’ or a variation of this term should be included in the title.)

Not applicable

Are sufficient details of the methods and analysis provided to allow replication by others?

Yes

Are the conclusions drawn adequately supported by the results presented in the review?

Yes

Reviewer Expertise:

Competency based Radiographic Education, Radiography and Imaging Technology, Image processing Techniques, Quality Assurance of Medical Imaging, Biophysics.

I confirm that I have read this submission and believe that I have an appropriate level of expertise to confirm that it is of an acceptable scientific standard.

## References

[1] : The potential for reduced radiation dose from deep learning-based CT image reconstruction: A comparison with filtered back projection and hybrid iterative reconstruction using a phantom. *Medicine (Baltimore)* .2021;100(19) : 10.1097/MD.0000000000025814 e25814 10.1097/MD.0000000000025814 34106619 PMC8133241

# Reviewer report: Reviewer response for version 1

**DOI:** 10.5256/f1000research.161532.r268958

**Published:** 2024-04-30

**Authors:** Shashi Kumar Shetty

**Affiliations:** 1 NITTE university, Karnataka, India

**Major comments:**

 The article provides an insightful and comprehensive review of the influence of Deep Learning Image Reconstruction (DLIR) on radiation dose, image noise, and outcomes in head and chest CT examinations compared to traditional Iterative (IR) and Filtered Back Projection (FBP) techniques. The methodology section is meticulously detailed, following PRISMA guidelines. The results section offers a clear summary of the inclusion and exclusion criteria, key outcomes related to DLIR's influence on radiation dose reduction, image noise reduction, and enhancement of image quality in CT head and chest examinations. The inclusion of quality assessment scores adds validation to the review's findings. The discussion and conclusion effectively explain the results, offering potential applications of DLIR in clinical settings. 

**Minor suggestions:**

 Please proofread for grammatical errors. Additionally, consider including points related to the smaller sample size of pediatric CT cases to provide a more comprehensive overview. 

**Suggestions to editor:**

 Overall, it is an excellent review article that represents a valuable contribution to the field of CT image reconstruction technology, providing insights into the impact of Deep Learning Image Reconstruction technology on dose reduction and improvement of image quality.

Are the rationale for, and objectives of, the Systematic Review clearly stated?

Yes

Is the statistical analysis and its interpretation appropriate?

Yes

If this is a Living Systematic Review, is the ‘living’ method appropriate and is the search schedule clearly defined and justified? (‘Living Systematic Review’ or a variation of this term should be included in the title.)

Not applicable

Are sufficient details of the methods and analysis provided to allow replication by others?

Yes

Are the conclusions drawn adequately supported by the results presented in the review?

Yes

Reviewer Expertise:

Radiation Biology, Radiation dose related research in CT, X-rays, Fluoroscopy etc.. Radiography.

I confirm that I have read this submission and believe that I have an appropriate level of expertise to confirm that it is of an acceptable scientific standard.
